# 

**Published:** 1879-12-20

**Authors:** 


### TABLE OF CONTENTS.

ORIGINAL AND SELECTED ARTICLES.

Introductory Address upon Southern Institutions, publicly delivered October 13, 1879, by T. S. Powell, M.D., 441. Treatment of the Drowned, 447. Cases of Impacted Cæcum and Colon, 449. Foreign Bodies on the Iris, 453. Treatment of Internal Hemorrhoids by hypodermic medication, 455.

ABSTRACTS AND GLEANINGS.

Laceration of the Perineum; Vaginitis; Carbuncle of the Urethra; Cancer of the Cervix, 457 Cystitis in the Female; Ovariotomy; Puerperal Fever, 458. Gathered Breasts; Treatment of the Funis; Congenital Syphilis, 459. Fissure of the Rectum; Habitual Constipation; Perimetritis; Diagnosis of Ovarian Cyst, 460. Miscellanea; Nasal Polypi, 461. Rhus Aromatica; Electricity in Amenorrhœa; Danger in the Use of Podophyllin, 462. Electricity in Impotence; Intra-Uterine Medication, 463. Plugging the Cervix Uteri for Metrorrhagia; Liquor Amnii, 464. Acute Nephritis; Injections of Water in Dysentery, 465. Treatment of Dysentery by Injections of a Solution of Chloral; Atropia in Croup; Moral Dietetics, 466. Notes from Practice; A Case of Amenorrhœa, 467. Narcotism from Nutmeg; Sanitas, the New Disinfectant; Boldo, 468. Singular Conflict of Opinion; Rag Weed as a Remedy for Rhus Poisoning: Buckeye in Chronic Rheumatism; Ice Cream and Beef Juice; Membranous Dysmenorrhœa, 469. First Insensibility from Ether; Eucalyptus in Bronchitis; Kolpoecpetasis *vs.* Kolpokleisis, 470.

SCIENTIFIC ITEMS.

Telephonic Phenomenon; Indestructibility of Matter; Diffusion of Gases, 471. Diffusion of Gases through Collodion Membrane; Valuable if True; Division of Labor Among Ants, 472.

PRACTICAL NOTES AND FORMULÆ.

Dysmenorrhœa; Chronic Rheumatism; Churchill's Tincture of Iodine; Good Formula for Administering Podophyllin, 473. Treatment of Dropsy; Post Nasal Catarrh; Sycosis, 474. Pavor Nocturnus; Sulphuric Acid in Cholera; Camphorated Dover's Powder; Anti-Asthmatic Powder; Syphiloderma, 475. Poison Oak Eruption; Erysipelas; To Prevent Malarial Fevers; Yerba Santa in Catarrhal Affections; Radical Treatment of Hernia; Cubebs in Whooping Cough, 476.

EDITORIAL AND MISCELLANEOUS.

Editorial Notices, 477. The Green-Eyed Phantom, 478. Book Notices, 479. Special Notices, 480.

